# Remote Ischemic Conditioning to Reduce Perihematoma Edema in Patients with Intracerebral Hemorrhage (RICOCHET): A Randomized Control Trial

**DOI:** 10.3390/jcm13092696

**Published:** 2024-05-03

**Authors:** Raviteja Kakarla, Gurpriya Bhangoo, Jeyaraj Pandian, Ashfaq Shuaib, Mahesh P. Kate

**Affiliations:** 1 Department of Neurology, Rangaraya Medical College, Kakinada 533003, India; kravicharles1988@gmail.com; 2 Faculty of Nursing, University of Alberta, Edmonton, AB T6G 1C9, Canada; gurpriya@ualberta.ca; 3 Department of Neurology, Christian Medical College, Ludhiana 141008, India; jeyarajpandian@hotmail.com; 4 Division of Neurology, Department of Medicine, University of Alberta, Edmonton, AB T6G 2G3, Canada; ashfaq.shuaib@ualberta.ca

**Keywords:** intracerebral hemorrhage, remote ischemic conditioning, perihematoma edema

## Abstract

**Background:** Early perihematomal edema (PHE) growth is associated with worse functional outcomes at 90 days. Remote Ischemic conditioning (RIC) may reduce perihematomal inflammation if applied early to patients with intracerebral hemorrhage (ICH). We hypothesize that early RIC, delivered for seven days in patients with spontaneous ICH, may reduce PHE growth. **Methods:** ICH patients presenting within 6 h of symptom onset and hematoma volume < 60 milliliters (mL) were randomized to an RIC + standard care or standard care (SC) group. The primary outcome measure was calculated edema extension distance (EED), with the cm assessed on day seven. **Results:** Sixty patients were randomized with a mean  ±  SD age of 57.5  ±  10.8 years, and twenty-two (36.7%) were female. The relative baseline median PHE were similar (RIC group 0.75 (0.5–0.9) mL vs. SC group 0.91 (0.5–1.2) mL, *p*  =  0.30). The median EEDs at baseline were similar (RIC group 0.58 (0.3–0.8) cm vs. SC group 0.51 (0.3–0.8) cm, *p*  =  0.76). There was no difference in the median day 7 EED (RIC group 1.1 (0.6–1.2) cm vs. SC group 1 (0.9–1.2) cm, *p*  =  0.75). **Conclusions:** Early RIC therapy delivered daily for seven days was feasible. However, no decrease in EED was noted with the intervention.

## 1. Introduction

Intracerebral hemorrhage (ICH) is a significant cause of morbidity and mortality worldwide. According to the Global Burden Disease estimates, in 2019, there were 3.4 million incidents of ICH cases and 2.8 million associated deaths [1]. The brain tissue adjoining the ICH is characterized by a progressive increase in water content and is defined as perihematomal edema (PHE). PHE growth is associated with increased disability and mortality both at 90 days (adjusted odds ratio (aOR), 1.17; 95% confidence interval (CI), 1.02–1.33 per 5 mL increase from baseline) [2] and at 6 months (OR: 1.60; 95%, CI: 1.04–2.46) [3]. In a prospective cohort study, a 10 mL increment in total lesion volume (ICH and PHE volumes) was independently associated with one-year death or poor functional outcomes (aOR: 1.24, CI: 1.11–1.42) [4]. PHE develops within a few hours after the onset of ICH [5], and its growth is most rapid within the first 24 h after ICH, with a relatively slower growth rate in the subacute period [3]. Recent studies have explored the relationship between the rate of PHE growth within the acute phase. Data from the Multicenter Risk Stratification and Minimally Invasive Surgery in Acute Intracerebral Hemorrhage (Risa-MIS-ICH) demonstrated that the initial PHE expansion (absolute increase in PHE volume from baseline to day 3) was associated with poor outcomes at 90 days in their population (*n* = 197, OR 1.049) after adjustment for hematoma volume [6]. PHE is a quantifiable marker of secondary brain injury following ICH; there is an unmet therapeutic need regarding the monitoring and control of PHE expansion. Adequate PHE control could serve as a potential clinical endpoint. Presently, there are no targeted management strategies for reducing PHE growth. Understanding the mechanisms of PHE’s growth may help plan applicable treatment strategies promptly.

The main event during the initial hyperacute phase includes clot retraction. The blood–brain barrier (BBB) permeability is not significantly impaired, and clot retraction causes an increase in interstitial osmotic pressure and changes to the trans endothelial Na+ gradient, which causes more fluid to be drawn into the extracellular space [7]. During this phase, aquaporin-4 (AQP4) causes an influx of extracellular water into the brain parenchyma to correct ionic imbalances, and inhibitors of AQP4 have demonstrated effectiveness in cytotoxic edema [8]. The acute stage involves disruption of the BBB, and a coagulation cascade is triggered, including the activation of thrombin and fibrinogen, leading to an increase in inflammatory mediators such as tumor necrosis factor-alpha (TNF-*α*), interleukin one beta (IL-1*β*), IL-6, IL-12, and intercellular adhesion molecule (ICAMs) [8,9]. There is also an increased expression of matrix metalloproteinase 2 and 9 (MMP-2, MMP-9), which mediates the degradation of the endothelial basement membrane and tight junctions, which can increase vascular permeability [9]. In the last phase of PHE development, approximately 72 h post-hematoma formation, resolution begins through erythrocyte lysis and phagocytosis. Erythrocyte lysis leads to hemoglobin accumulation, contributing to neuronal damage. The intraparenchymal presence of lysed red blood cells increases BBB permeability without enhancing cerebral blood flow, indicating that vascular dysfunction is also at play [10]. Given the evolution of PHE, strategies targeting specific mediators will likely result in improved patient outcomes.

PHE volume and growth assessment has been challenging due to its dependence on hematoma volume. PHE is further influenced by the location of the hematoma, supratentorial versus infratentorial and cortical versus subcortical location [11]. The edema extension distance (EED) represents the average thickness in centimeters of the edema beyond the boundary of the hematoma. It is independent of the hematoma volume (HV) [12]. Wu et al. found that an unexpected EED within 72 h of onset was associated with a 6-month mortality rate [3]. Recognizing its independence from the hematoma volume, which is variable, this theoretical model was tested by using the patient data from the conservative arms of MISTIE 1 and the INTERACT trial and compared them with the conventional edema measures and found that using EED as an clinical outcome measure reduced the sample size requirements by as much as 75% [12]. 

The current standard of care focuses on blood pressure control, and there is a lack of clearly defined protocols specifically addressing PHE control. Reduction in systolic blood pressure (BP) has shown to be effective for hematoma management; however, the effects on PHE have been mixed. The INTERACT trials showed that there was no difference in the adjusted mean absolute increases in edema volumes at 24 and 72 h during intensive reduction of BP (target systolic BP 140 mm Hg) versus a standard guideline-based management of BP (target systolic BP 180 mm Hg) (95% CI, −0.45 to 5.22; *p* = 0.10) [13]. An analysis of the ATACH-2 trials showed that in a subset of 780 patients, intensive BP reduction caused a decrease in the PHE expansion rate during the first 24 h period in a multivariable (β = −0.12; *p* = 0.03) analysis [13,14]. The INTERACT-3 trials used a bundled care approach towards spontaneous ICH, which included lowering of systolic blood pressure (target < 140 mm Hg), glucose control (target 6.1–7.8 mmol/L in those without diabetes and 7.8–10.0 mmol/L in those with diabetes), anti-hyperthermic treatment (target body temperature ≤ 37.5 °C), and reversal of oral anticoagulants (target international normalized ratio < 1.5) within one h of treatment, in patients who presented with these disturbances [15]. Glycemic control may be necessary when examining PHE, as hyperglycemia may impair capillary integrity [6]. Temperature reduction in the first 24 h following ICH was independently associated with a good functional outcome at three months (aOR, 11.28; 95% CI, 4.69–27.01; *p* < 0.0001) within this sample [16]. The literature rationalizes that temperature-lowering measures inhibit inflammatory factors contributing to cerebral edema following an ICH [17]. Anticoagulant therapy is linked to greater hematoma expansion upon spontaneous ICH and, by extension, lower mean relative PHE and mean EED; however, a recent study showed that, for individuals presenting with oral anticoagulant-associated intracerebral hemorrhage, the absolute PHE was not different [18]. 

Remote ischemic conditioning (RIC) is a simple, inexpensive, and safe intervention in which the repetitive inflation of a blood pressure cuff on a limb may protect distant organs after an ischemic injury. RIC is being studied actively in the treatment of ischemic stroke to reduce infarct growth and has been beneficial within pre-clinical models with regards to inducing angiogenesis, vasodilation, and activating anti-inflammatory pathways. Given RIC’s ability to bolster collateral circulation, it may play a protective role when there are multiple infarcts present. A recent trial included the remote ischemic conditioning combined with intravenous thrombolytics for acute ischemic stroke (SERIC-IVT), and they found that patients receiving RIC had a lower level of high sensitivity-C reactive protein compared with the control group (*p* = 0.048) [19]. Furthermore, the remote ischemic conditioning after stroke trial 2 (RECAST 2) indicated an increase in S100ß protein within the sham group (mean rise 111 pg/mL, *p* = 0.041) but not the RIC group. S100ß is a recognized surrogate marker of brain injury severity and may be released into the bloodstream following ischemic, hemorrhagic stroke or other traumatic brain injuries [20]. Given that inflammation is common to both acute ischemic and hemorrhagic stroke, RIC may have the potential to target inflammatory pathways and extend utility within the treatment of ICH and PHE management.

Recently, animal studies have shown improvement in hematoma resolution after applying RIC. Mixed-sex C57BL/6J littermates (eight per group) were randomized to receive once-daily mock conditioning or bilateral RIC beginning at two hours after sham or collagenase-induced ICH. RIC reduced hematoma volume by 43% (improved cerebral blood flow by 24%) compared to sham-conditioned mice after ICH on day five after injury [21]. Another animal study was conducted in Sprague Dawley rats by injecting collagenase to induce ICH. RIC therapy was applied by compressing and releasing the bilateral femoral arteries using aneurysm clips for three cycles (each occlusion or release for 10 min). The intervention did not worsen hematoma volumes or brain edema compared to the controls [22]. Research within animal models has demonstrated that RIC can target the molecular mechanisms proposed in the evolution of PHE. RIC can downregulate inflammatory cytokines such as IL-1β, IL6, and TNF-α and suppress hypoxia-inducible factor 1 alpha (HIF-1α) [23,24]. Within rodent models, these cytokines have been associated with the upregulation of AQP4; RIC can target cytokine overexpression and control inflammation and parenchymal edema [25]. Furthermore, there is also a relationship between RIC and MMP-9 attenuation. In adult male Sprague Dawley rats, animals receiving RIC showed a 58% reduction in the MMP-9 expression relative to controls [26]. Lastly, RIC has also been shown to enhance the activation of adenosine monophosphate-activated protein kinase (AMPK) in C57BL/6J mice, which served as a molecular switch to initiate the anti-inflammatory functions for circulating myeloid cells [21]. RIC has been shown to accelerate CD36-mediated phagocytosis of lysed red blood cells within this model [21]. 

The treatment of ICH presents this two-pronged issue: there needs to be a way to manage the initial hematoma but also maintain continued care after stabilization to prevent cerebral edema. RIC has demonstrated the ability to tackle both issues in preclinical models and may warrant a thorough investigation to understand the biochemical and physiological effects on humans. Pilot studies have demonstrated some beneficial effects; for example, a study conducted in human volunteers showed a dynamic change in cerebral autoregulation 6 h after the onset of RIC. The study also showed increased vascular endothelial growth factor (VEGF) after 1 h of RIC in healthy subjects and increased anti-inflammatory cytokines when studied simultaneously [27]. The aim of this study is to examine the feasibility of early RIC and measure its effect on PHE growth over a 7-day period. Given the potential anti-inflammatory properties of RIC, we hypothesized that RIC would reduce the PHE as assessed by EED at seven days in patients with acute ICH compared to the standard of care.

## 2. Methods

### 2.1. Patients

The remote ischemic conditioning to reduce perihematomal edema in intracerebral hemorrhage patients (RICOCHET) trial was a prospective randomized, controlled, single-center, interventional blinded endpoint study with 1:1 allocation of intervention or standard of care. Eligible patients were ≥18 years of age and had first-ever stroke symptoms with a spontaneous ICH diagnosed on non-contrast computed tomography (CT) < 6 h after onset. These patients presented to the neurology office of the Christian Medical College and Hospital Ludhiana, India. Patients with evidence of a secondary ICH (e.g., vascular malformation), parenchymal hematoma volume (PHV) ≥ 60 mL on baseline head CT, coagulopathy or platelet counts less than 2 × 10^5^/microliter, injury or easy bruising of the upper arm skin due to underlying skin condition, pain or tenderness in the upper arm muscles, fever on admission, sulfonylurea medication for diabetes control, and known upper arm peripheral arterial disease were excluded. The patient or surrogate decision-maker provided informed consent. The protocol was approved by the local human research ethics committee.

Baseline demographic information and medical history were abstracted. The National Institutes of Health Stroke Scale (NIHSS) score and Glasgow Coma Scale (GCS) were assessed at admission, at 24 h, and on day seven or the day of discharge. Blood pressures were recorded on admission for 5 min, then hourly for 24 h, and then daily until the day of discharge. The functional outcome was assessed at 24 h, day 7, and 90 days with the modified Rankin Scale (mRS).

### 2.2. Randomization

Patients were randomized 1:1 using block randomization with an online randomization service (Random.org) into either intervention or control groups. Qualified investigators were blinded to the treatment assignments and assessed CT scans and neurological outcomes.

### 2.3. Study Intervention 

RIC was delivered in two sessions per day in both arms for seven days and up to the time of discharge, whichever was earlier. Each cycle consisted of 5 min of pressure (ischemia) and 5 min of relaxation (reperfusion). The pressure was increased by 30 mm Hg above the systolic pressure in the upper arm or to a maximum of 200 mm Hg. The patients received four cycles per session. The intervention was delivered with the help of a standard brachial blood pressure cuff. The intervention was delivered by study personnel (RK). 

All patients in the control group received standard care with blood pressure management and stroke unit care. To briefly discuss the standard of care, blood pressure control with the systolic BP was kept under 140 mm Hg within the first hour. Furthermore, all patients were admitted to the stroke unit, where they received deep vein thrombosis (DVT) prophylaxis, fever, blood sugar, and swallowing (FeSS) management.

### 2.4. Image Acquisition

All CT scans were performed using a Philips Ingenuity 128 slice CT Machine (Amsterdam, The Netherlands) and consisted of 4.8 mm sections (120 KV (peak), 200 mA per section) through the entire brain (27 ± 33 sections with a 512 × 512 matrix). Patients with a hematoma volume ≥ 60 mL were excluded by calculating the CT scan hematoma volume through the ABC/2 method. A repeat CT scan was performed after 24 h and on the seventh day or the day of discharge as part of the standard of care and for assessing hematoma volume. Interval CT scans were also performed if necessary if there were signs of neurological worsening.

### 2.5. Imaging Analyses

The intra-parenchymal, intraventricular, and perihematomal volumes in patients with ICH were analyzed and measured using AnalyzePro v.1.0 software (AnalyzeDirect, Overland Park, KS, USA). 

**Figure 1:**
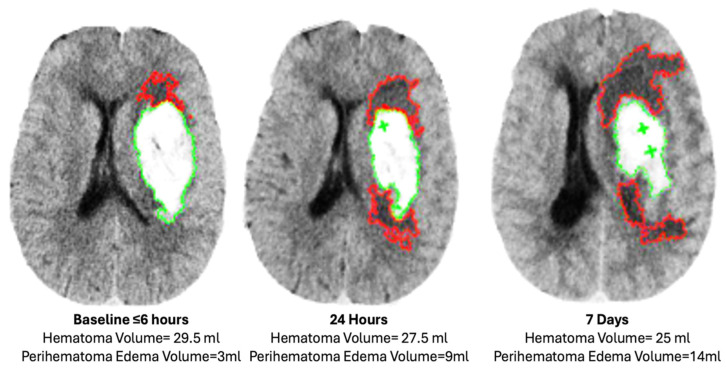
Hematoma and perihematoma volume measurement with planimetric technique. Green outline indicates hematoma identified based on the high Hounsfield units. Red outline indicates perihematomal edema identified on the basis of the low Hounsfield units.

Intraparenchymal volume (IPHV) and Intraventricular hemorrhage volume (IVHV) were assessed using semi-automatic and manual planimetric methods. For the segmentation of ICH, the Housenfield unit (HU) range was kept within 44 to 100 HU. The visibly hypo-dense region was manually outlined, and a threshold of 5 to 23 HU was used to objectively define perihematomal edema volumes (Figure 1) [28]. Relative edema was calculated as the ratio of this PHE volume to total hematoma volume. The edema extension distance (EED) was calculated on the three scans. The EED represents the thickness of the edema around the hematoma and is measured in centimeters. EED is independent of hematoma volume. The formula for EED [12] is

$$\sqrt[{3}]{\frac{Edema\;Volume+ICH\;Volume}{1.33}}-\sqrt[{3}]{\frac{ICH\;Volume}{1.33}}$$

### 2.6. Outcome Measures

The primary outcome measure was the calculated EED at seven days. The secondary imaging outcome measure was the hematoma growth volume at 24 h and seven days, absolute PHE volume at 24 h and seven days, and relative PHE volume at 24 h and seven days. According to previous studies, hematoma expansion or growth volume was defined as an absolute increase in the hematoma volume of more than three mL [28]. The secondary clinical outcome was mRS at day 90.

### 2.7. Sample Size

According to the Helsinki ICH study, the expected EED after ICH can be calculated with the following formula: expected EED = 2.210 × (Days)^0.07331^ − 1.478. Thus, the expected EED at seven days is 1.0708 ± 0.23 cm [3]. We hypothesized that RIC would reduce the calculated EED by 21% to a mean of 0.84 cm. The sample size required to test the hypothesis is 16 patients in each group for a total of 32 patients for alpha of 5% and power of 80%. Due to financial constraints, many patients leave the hospital against medical advice at our center [29]. Patients did not have to pay for the study intervention. In a previous study conducted at the center, 50% of patients left the hospital against medical advice [29]. To account for the dropout rate, we planned to enroll 30 patients in each group for a total of 60 patients.

### 2.8. Statistical Analyses

Continuous variables, including but not limited to age, onset to randomization (minutes), systolic and diastolic blood pressure, and heart rate, were expressed as means and standard deviation. 

NIHSS, IPHV, absolute PHE, relative PHE, EED, and length of stay in the hospital were not normally distributed and were expressed as median with interquartile range (IQR). Nominal variables like sex, presence of risk factors, and death were expressed in numbers and percentages. The group means were compared with unpaired *t*-tests, and the medians were compared with the Mann–Whitney U test. All patients were analyzed according to intention to treat analysis. The nominal variable within the groups was compared with the chi-squared test, and if the number of patients in each group was <5, the Fischer’s exact test was applied. The study’s primary outcome was a continuous variable, and group medians were compared with the Mann–Whitney U test. The significance level was set at *p* < 0.05. All statistical analyses were performed using SPSS version 21 (IBM Corp.,Armonk, New York, NY, USA). 

The data collected in this study are available upon request. Requests for access to the dataset should be directed to the corresponding author. 

## 3. Results

### 3.1. Baseline Characteristics

**Figure 2:**
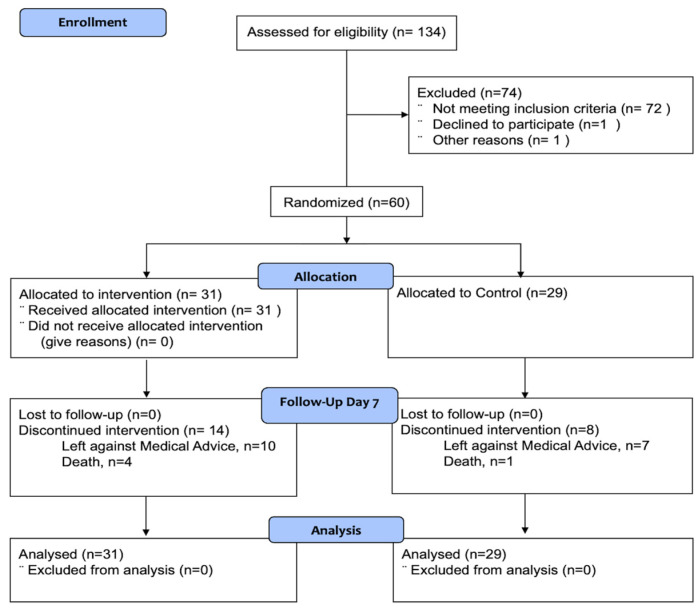
Consort figure.

A total of 60 patients or a surrogate consented and were randomized, of whom 31 received the intervention, and 29 were treated as the control group (Figure 2). The study groups had no baseline difference in demographic, clinical, and imaging characteristics (Table 1). The baseline perihematomal volume, relative perihematomal edema, and EED measures were comparable. 

### 3.2. Blood Pressure Control

**Figure 3:**
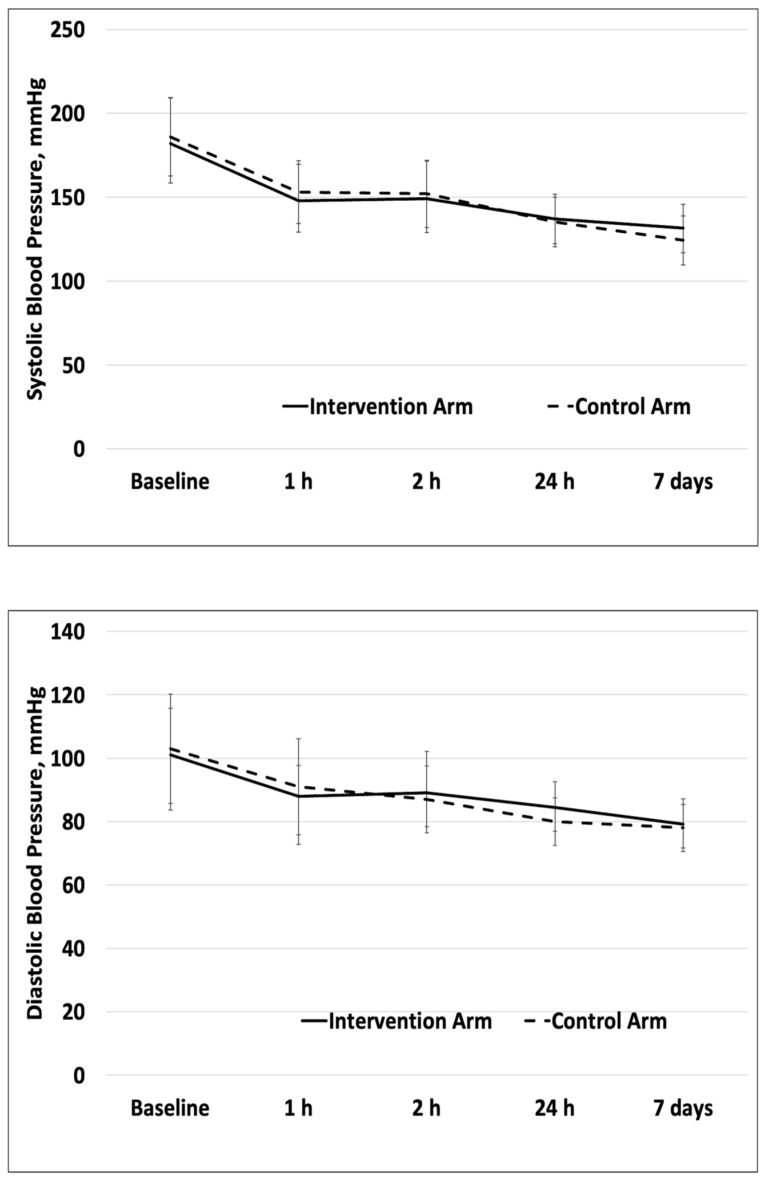
Blood pressure control in intervention and control group during the study period.

The mean systolic BP at baseline was 182 ± 27.2 mmHg and 186 ± 23.4 mm Hg in the intervention arm and control arm, respectively (*p* = 0.5) (Table 1). One hour after randomization, the systolic BP decreased in both groups to 148 ± 21.6 mm Hg and 153 ± 18.7 mm Hg in the intervention and control groups, respectively (*p* = 0.4) (Figure 3). The systolic BP at 24 after randomization was 136.9 ± 14.8 mm Hg and 135.3 ± 14.7 mm Hg, respectively (*p* = 0.7). The systolic BP at seven days was 131.5 ± 14.4 mm Hg and 124.4 ± 14.6 mm Hg, respectively (*p* = 0.2). The mean baseline diastolic BP was 101 ± 14.74 mm Hg in the intervention group and 103 ± 17.31 mm Hg in the control group. An hour after randomization, the diastolic BP decreased to 88 ± 9.75 in the intervention and 91 ± 15.19 in the control group (*p* = 0.47).

### 3.3. Fidelity of RIC

The maximum number of sessions that could be delivered to a patient was 14 for a total duration of 7 days or until the date of discharge, whichever was earlier. The median sessions delivered to the patients in the intervention group was 12 (IQR 3–14). For the intervention arm, out of the original 31 patients, only 58.1% of intervention patients could continue until day 4 (*n* = 18), and 12 patients (38.7%) could continue until day 7. The dropout rate was 41.9% by day 4 and 58.1% by day 7. The most common reason for discontinuation was that 32.2% (*n* = 10) left the hospital against medical advice in the intervention group, whereas 24.1% left against medical advice in the control group (*n* = 7), 12.9% in the intervention group [4] died, and 9.6% had stopped the intervention because of pain during the procedure (*n* = 3).

### 3.4. Outcome Measures

At baseline, the median EED calculated for 31 patients in the intervention group and 29 patients in the control group was 0.58 cm (0.34–0.79 cm) and 0.51 cm (0.29–0.79), respectively (*p*= 0.7) (Table 2). The median EED calculated at 24 h based on 27 patients in the intervention and 23 patients in the control group was 0.77 cm (0.52–0.95) and 0.64 (0.51–0.84), respectively. On day seven, based on 16 patients in the intervention arm and 21 patients in the control group, the median EED was 1.10 (0.59–1.17) and 1.03 (0.89–1.19), respectively (*p* = 0.7). Regarding the expected EED (cm) on day seven of discharge, the expected EED on day 7 was 1.07 cm. The patients who had a higher-than-expected EED (*n* = 16) was 9 (56.2%) in the intervention group and 7 in the control group (*n* = 21) (33.3%, *p* = 0.3). Seven patients had lower than expected EED in the intervention group (43.8%) compared to fourteen patients in the control group (66.7%). 

From baseline to 24 h, 29% in the intervention group (*n* = 8) had hematoma expansion, whereas 13% in the control group (*n* = 3) had hematoma expansion. From 24 h to day 7, 12.5% of patients in the intervention group (*n* = 2) and 9.5% in the control group had hematoma expansion. By 24 h and seven days, there was no difference between the absolute and relative PHE between the intervention and control arms (the functional outcome of the patients at day 90). The follow-up at 90 days was available for 30 patients in the intervention group (96.7%) and 28 in the control group (96.5%). There was no difference between good outcomes (mRS 0–2) between the intervention and control arms (*p* = 0.2). Mortality was also similar in the intervention and control groups (33.3% vs. 32.1%, *p* = 0.2).

## 4. Discussion

Our study noted no difference in EED between the RIC and control arm at seven days. However, at seven days, a higher-than-expected EED was observed in 44.7% of patients in our cohort. Absolute and relative PHE growth was similar in both groups. Both study groups had similar systolic and diastolic BP control at 1 h, 24 h, and seven days. 

In the remote ischemic conditioning for intracerebral hemorrhage trial (RICH-1), 40 participants presenting with symptoms of supratentorial intracerebral hemorrhage received RIC along with medical therapy for seven days. The hematoma resolution rate in the RIC group (49.25  ±  9.17%) was higher than in the control group (41.92  ±  9.14%; *p*  =  0.015). Also, the relative PHE in the RIC group (1.77  ±  0.39) was lower than in the control group (2.02  ±  0.27; 95% *p*  =  0.023) [30]. Our study did not observe a change in the RIC group’s hematoma or PHE volumes. Several transitional challenges limit the consistency of the RIC’s effect on humans. The animal models used within RIC trials lack the comorbidities present in humans, which could attenuate the effect of the therapy. Diabetes mellitus, hypothyroidism, hypertension, hematological disorders, and smoking can reduce RIC efficacy. Vascular dysfunction is a common occurrence in individuals with diabetes, and the presence of these impairments can blunt the benefits offered by RIC [31]. Our study excluded patients taking sulfonylureas, as these drugs may mask the effects of RIC. However, diabetes can lead to decreased endothelial nitric oxide synthase (eNOS) activity and an increase in pro-inflammatory pathways [31]. A larger dose of RIC may have been required to elicit an effect within the majority of our intervention arm to overcome the physiologic dysfunction. Furthermore, dyslipidemia has had varying effects on the effect of RIC. In a post-hoc analysis of the 1419 patients in the RICAMAS study, it showed that a significantly higher proportion of the modified Rankin scale score 0 to 1 was identified in the RIC versus control in the high-total cholesterol group (62.8% vs. 55.5%; *p* = 0.059) and high-triglycerides group (67.8% vs. 60.1%; *p* = 0.099), indicating that blood lipids had no impact on the neuroprotective effects of RIC in their sample population [32]. In contrast, another study consisting of 24 patients observed that RIC protected from endothelial dysfunction induced by forearm ischemia-reperfusion in healthy controls (flow-mediated vasodilation (FMD) baseline 2.8  ±  2.3 vs. FMD after I/R + RIC 4.5  ±  4.0%; means SD) and in patients with familial hypercholesterolemia (FH) with low LDL-C (4.5  ±  3.5 vs. 4.4  ±  4.2%). However, RIC was ineffective in patients with FH and high LDL-C (FMD 3.9  ±  3.0 vs. 1.1  ±  1.5%; *p* < 0.01) [33]. In addition, statins are also known to blunt the effect of RIC, and this may have further attenuated the effects in the intervention arm [34]. Given the presence of the comorbidities and the strict blood control within the control arm, we may have been unable to detect a difference. Another important implication within treatment for ICH may include the presence of hematological disorders. Cerebral hemorrhage can serve as the presenting manifestation of a hematological disorder, and the underlying disorder may need to be addressed as a treatment consideration for the ICH [35]. Hematological disorders found within a study examining the relationship between cerebral hemorrhage and hemodynamic function included essential thrombocythemia, polycythemias, subtypes of multiple myeloma, and leukemia. In the case of hemodynamic impairment, blood pressure control fails to address the underlying cellular impairments, and an investigation into the patient’s hematological profile may yield merit. Age is a risk factor for stroke in humans, and it plays a role in the recovery period following a stroke, which is often a factor not accounted for in translational research, as much of the preclinical research is conducted on young rodents (8–12 weeks old) [36]. 

The second factor affecting the translation may be the timing of RIC intervention [37]. Many animal studies focus on remote ischemic preconditioning, which is relevant when ischemia is expected, i.e., myocardial ischemia during cardiac or vascular surgery. However, given the unpredictable nature of stroke, per- or post-conditioning may be more feasible within clinical trials as patients present with sudden acute ischemic stroke or spontaneous ICH. The time to RIC may vary between animal and human models. In the RICAMIS trial, ischemic stroke patients who had early RIC initiation within 24 h of onset experienced better clinical outcomes than those who received RIC between 24 and 48 h [32]. On the contrary, in the RICH-1 trial, RIC intervention was delivered to patients with ICH in the first 24–48 h. Interventions targeting hematoma expansion, such as BP reduction and rFVIIa, may have to be implemented in the first 2–6 h [38]. Interventions targeting inflammation, like RIC and colchicine, may have to be implemented within 24 h [39].

The third factor affecting the translation may be the dose and duration of RIC treatment; preclinical studies use 3–10 cycles of ischemia/reperfusion lasting between 5–15 min, delivered in the lower limb via femoral artery occlusion. However, human studies use 4–5 cycles lasting 5 min each in the upper limb with a BP cuff [40]. Furthermore, if we target PHE growth, it can occur in two phases, early and delayed. Delayed PHE growth is also associated with worse functional outcomes. The Chinese Intracranial Hemorrhage Image Database (CICHID, *n* = 312) showed that, on average, the growth peak of PHE occurred 14 days after ICH onset. An absolute PHE increase from 4 to 7 days to 8–14 days by 3.3 mL was associated with poor functional outcome. Additionally, an increase in PHE by 3.8 mL from 8 to 14 days to 15–21 days was associated with poor functional outcomes. Delayed PHE occurred in approximately 40% of patients in that cohort and was associated with larger baseline hematoma volume [41]. This RIC may have to be administered for 21 days instead of 7 days. 

The fourth factor affecting translation could be good early BP control in both study groups. We postulate that the rigorous blood pressure control under the standard care procedure may have preemptively restricted hematoma growth and PHE, as these two processes are linked. Several large-scale clinical trials have demonstrated that early blood pressure control has been linked to reduced hematoma growth and favorable functional outcomes. A post-hoc analysis of the ATACH-2 trials looked at intensive blood pressure reduction with nicardipine within the first 2 h of symptom onset. The attained BP in the treatment arm was 120.5  ±  13.9 mm Hg vs. 140.6  ±  16.7 mm Hg in the standard treatment group (*p*  <  0.001) less than 2 h after onset of symptoms. Associated with this ultra-early intensive blood pressure control was a lower risk of hematoma growth (OR, 0.56; 95% CI, 0.34–0.92; *p* = 0.02) [42]. Similarly, within our study, we were able to decrease systolic pressures from 182 ± 27.23 mm Hg (intervention) and 186 ± 23.42 (sham) (*p*= 0.513) at baseline to 149 ± 22.47 mm Hg and 152 ± 19.96 mm Hg (*p* = 0.638) by the second hour in the intervention and control arms, respectively. Given that the blood pressure control was similar within the groups, this may have acted as a confound and dampened the effects of the RIC. 

Our study had limitations; the sample size was small, with 30 patients in each group. Furthermore, there was a dropout rate of 42% by day 4 and 58% by day seven; 32% of individuals left against medical advice due to financial constraints. Indian healthcare includes public and private care; however, there are significant disparities in funding at these levels [43]. A large proportion of Indians are paying out-of-pocket for healthcare expenses, and this contributes to leaving against medical advice, which was the case for our present study. One in eleven individuals stopped the RIC treatment due to pain or discomfort. Nevertheless, no serious adverse events were noted. Dropout rates in acute ICH clinical trials must be accounted for in future trials. We expected a 21% absolute reduction in EED. The RIC effect in ICH may be small and multipronged, including hematoma resolution, decreased PHE growth, and reduced inflammation. In our study a 90-day outcome was assessed via telephone; thus, cause of death could not be ascertained. 

Future randomized clinical trials may have to consider the minimization for diabetes and change in the delivery of intervention (bilateral arm versus lower limb), RIC delivery, as it may affect the efficacy of RIC. Two ongoing studies will help us understand the utility of RIC in ICH. The remote ischemic conditioning for intracerebral hemorrhage (RICH-2) is a phase 3 clinical trial assessing the effect of RIC on functional outcomes at 90 days. Four hundred and fifty-two patients with a supratentorial ICH volume of 10–30 mL within 24–48 h of symptom onset with a moderate neurological deficit will be randomized to receive daily RIC in one arm for seven consecutive days or sham RIC treatment. The safety and efficacy of remote ischemic conditioning in patients with spontaneous intracerebral hemorrhage (SERIC-ICH) is another prospective, multicenter, randomized controlled, double-blind trial aimed at examining RIC treatment and its corresponding effect on functional outcomes at 180 days in patients with ICH [44]. A total of 2000 patients with supratentorial ICH with a moderate neurological deficit will be randomized to receive RIC twice daily for seven days. 

## 5. Conclusions

Adjunct RIC treatment in acute or subacute ICH may enhance endogenous hematoma absorption and reduce PHE growth. In our study, we did not observe the effect of twice daily RIC in both arms on EED at seven days. RIC may have a smaller effect size, so a larger sample size study needs to be planned. 

## Figures and Tables

**Table 1:** Baseline demographic clinical and imaging characteristics.

	Intervention (*n* = 31)	Control Arm (*n* = 29)	*p*-Value
Age (mean ± SD), years	59 ± 10.23	56 ± 11.40	0.355
Female Sex *n* (%)	9 (29.0)	13 (44.8)	0.205
Vascular Risk factors			
Hypertension, *n* (%)	31 (100.0)	28 (96.6)	0.483
Diabetes, *n* (%)	5 (16.1)	8 (27.6)	0.355
Dyslipidemia, *n* (%)	17 (54.8)	18 (62.1)	0.570
Smoking, *n* (%)	4 (12.9)	4 (13.8)	0.919
Coronary artery disease, *n* (%)	31 (100.0)	28 (96.6)	0.483
Alcohol, *n* (%)	5 (16.1)	8 (27.6)	0.355
Timing			
Onset to arrival (min) median (IQR)	90 (60–150)	60 (30–210)	0.682
Onset to randomization (min) median (IQR)	210 (170–270)	180 (92–300)	0.471
Clinical Variables			
SBP (mm Hg) baseline	182 ± 27.23	186 ± 23.42	0.5
DBP (mm Hg) baseline	101 ± 14.74	103 ± 17.31	0.6
HR (per minute) baseline	90 ± 11.46	87 ± 11.28	0.3
Glasgow Coma Score (GCS)	12 ± 2.86	12 ± 3.15	0.9
NIHSS Median (IQR)	19 (13–23)	18 (13–21)	0.2
ICH Location			
Supratentorial Deep *n* (%)	29 (93.5)	24 (82.8)	
Cortical *n* (%)	1 (3.2)	2 (6.9)	0.481
Infratentorial *n* (%)	1 (3.2)	3 (10.3)	
Only IPH	20 (64.5)	16 (55.2)	0.460
IPH + IVH	11 (35.5)	13 (44.8)	

IPH: intraparenchymal hemorrhage; IVH: intraventricular hemorrhage.

**Table 2:** Intracerebral hemorrhage and perihematomal edema evolution over seven days.

Imaging Parameters Median (IQR)	At Baseline			At 24 H			At 7 Days		
	Intervention (*n* = 31)	Control (*n* = 29)	*p*	Intervention (*n* = 27)	Control (*n* = 23)	*p*	Intervention (*n* = 16)	Control (*n* = 21)	*p*
IPH vol. (mL)	14 (7–27)	10 (6–15)	0.1	15 (8–29)	10 (7–18)	0.1	12 (8–18)	12 (8–18)	0.3
IVH vol. (mL)	0 (0–7)	0 (0–9)	0.6	0 (0–8)	0 (0–7)	0.9	0 (0–1)	0.5 (0–4)	0.3
TH vol. (mL)	21 (8–30)	13 (8–28)	0.2	20 (10–37)	17 (9–26)	0.1	14 (8–18)	14 (11–24)	0.5
Absolute PHE vol. (mL)	14 (7–26)	10 (3–21)	0.3	22 (10–41)	14 (9–26)	0.1	33 (10–46)	29 (20–38)	0.3
Relative PHE vol. (mL)	0.75 (0.52–0.95)	0.91 (0.54–1.24)	0.3	1.20 (0.92–1.44)	1.33 (0.82–1.67)	0.7	1.99 (1.29–2.67)	1.95 (1.44–3.06)	0.6
EED (cm)	0.58 (0.34–0.79)	0.51 (0.29–0.79)	0.8	0.77 (0.52–0.95)	0.64 (0.51–0.84)	0.3	1.10 (0.59–1.17)	1.03 (0.89–1.19)	0.7

IQR, interquartile range; IPH, intraparenchymal hematoma; Vol., volume; IVH, intraventricular hematoma; TH, total hematoma; PHE, perihematoma edema; EED, edema extension distance.

## Data Availability

Data will be available to interested researchers, please contact the corresponding author.
